# Importance and methodologies of endodontic microleakage studies: A systematic review

**DOI:** 10.4317/jced.53604

**Published:** 2017-06-01

**Authors:** Farnaz Jafari, Sanaz Jafari

**Affiliations:** 1 Assistant Professor, Department of Endodontics, Dental School, Tabriz Branch, Islamic Azad University; 2 Assistant Professor, Orthodontics Department, Dentistry Faculty, Ilam University of Medical Science.

## Abstract

**Introduction:**

It is very important to obtain a tight seal in obturated root canal, making it necessary to conduct clinical or laboratory studies on the sealability of endodontic materials. Different methodologies have been historically used to assess microleakage of different endodontic materials. The aim of the present study was to comprehensively review different material testing methods used in microleakage studies, their interpretation and importance in endodontic literature.

**Material and Methods:**

A systematic search was conducted in Medline, Cochrane, and Web of Science databases. In addition, the reference lists of review articles on the topic were searched. No language restriction was applied. Two independent reviewers screened the article.

**Results:**

Microleakage is considered the single most important risk factor responsible for apical periodontitis. Dye penetration, dye diffusion, bacterial and endototoxin infiltration, fluid filtration, glucose, caffeine and protein infiltration, radioisotope penetration, animal studies, and electrochemical or 3D evaluation are different methodologies used to assess dental leakage. 91 out of 177 articles in the primary search were included in the study. These methods are very divergent in their viewpoints; that is why their results cannot be easily compared. It is necessary to standardize microleakage detection methods in order to more correctly evaluate the phenomena that are found between the root canal wall and the root canal filling materials.

**Conclusions:**

All the methods are useful if studies are performed strictly with large sample sizes and proper control groups and if the technique can be standardized. Furthermore, more evaluations of the reliability of the methods are strongly recommended.

** Key words:**Dental leakage, review, root canal, material testing methods, data interpretation.

## Introduction

The three chief aims of obturation are: 1) To entrap bacteria remaining in the root canal system; 2) To prevent the ingress of periapical tissue-derived fluid into the root canal; and 3) To prevent coronal leakage of bacteria (1).

New root filling materials are developed at an attempt to improve the ability and efficacy to eliminate infections and prevent re-contamination (2). Apical periodontitis is not now believed to be caused by stagnation of fluid within unfilled root canal spaces (3); rather, it is believed to be an inflammatory reaction of periapical tissues and an immunologic response of the host defense system to microorganism contaminating the root canal system (4,5). As a result, the success of root canal therapy critically relies on elimination of pulp space infection and contamination (6-8). Root canal infection, in a manner similar to many other infections, is mainly a biofilm-induced condition (9,10). A relationship has been established between bacterial biofilms and induction of apical periodontitis by many recent histo-bacteriological studies (11,12).

Extra-radicular infection, including apically extruded dentin debris in association with bacteria lodged in dentinal tubules, true radicular cysts, and foreign body reactions require surgical interventions (13).

The present review addresses the importance of microleakage in endodontics and methodologies used in endodontic microleakage studies.

## Material and Methods

**Figure 1:**
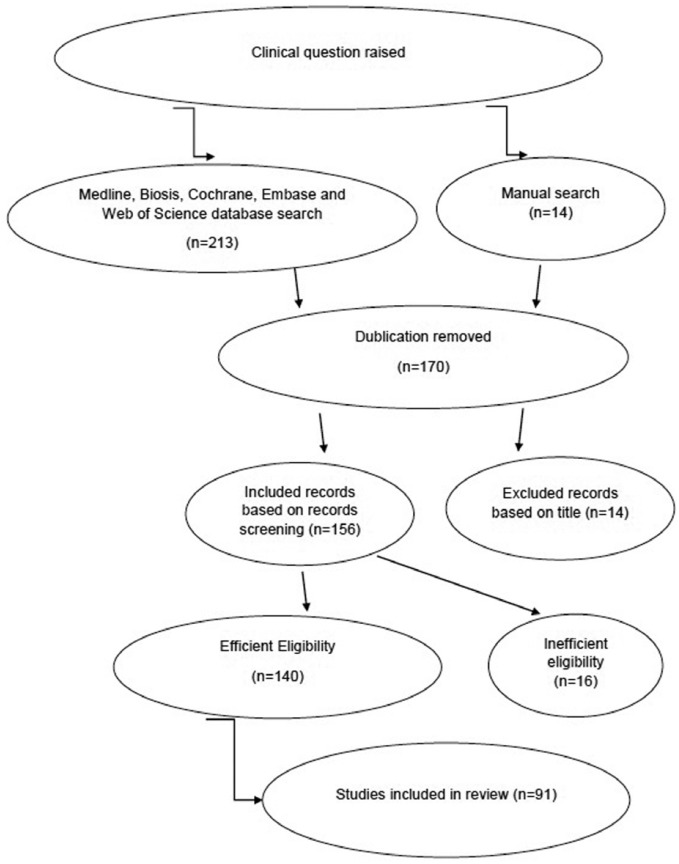
PRISMA Flow Diagram for selected articles.

A systematic search was run in Medline, Biosis, Cochrane, Embase, and Web of Science databases. In addition, the reference lists of review articles on the subject were searched. No language restriction was implemented. Two independent reviewers screened the articles. Quality assessment of the included articles performed by number of citations of the articles, methodology assessment, criticism on the articles’ methodologies, detailed report of the results and consistency between results and discussion part. Selected articles thoroughly studied and main topics included in the study. PRISMA flow diagram (14) for selected articles demonstrated in figure 1.

-Inclusion and exclusion criteria

Peer-reviewed journals were searched for studies on endodontic microleakage and sealability of endodontic materials published until June 2016. Studies that did not meet these inclusion criteria were excluded.

-Search methodology

Appropriate MeSH headings and key words related to the importance of sealability and different methodologies used to evaluate endodontic microleakage were used to run searches in PubMed/Medline, Biosis, Cochrane, Embase, and Web of Science databases.

Moreover, a hand search was carried out in the last 2 years of issues of the following endodontic journals: International Endodontic Journal; Journal of Endodontics; Oral Surgery, Oral Medicine, Oral Pathology, Oral Radiology and Endodontology; Brazilian Journal of Endodontics and Journal of Dental Materials. Cross-referencing process continued until no new articles were identified. Keywords included dental leakage, root canal, material testing methods, data interpretation, importance, microleakage, dye microleakage, endodontics, protein leakage, glucose leakage, fluid filtration, dye extraction, bacterial leakage, endotoxin microleakage, protein microleakage, radio-isotope, animal study, scaning electron microscope and cone beam computed tomography.

This primary search yielded 177 papers until June 2016, 86 were excluded due to inconsistency to the topic. The remaining 91 met authors’ inclusion criteria and were included in this study. PRISMA flow diagram (14) for selected articles stated in figure 1.

## Results

-Importance

One of the goals of root canal obturation is to obtain a hermetic seal of the root canal system to induce apical and periapical healing after endodontic treatment (15). Inadequate filling and obturation might result in penetration of fluids into the voids, inducing a periapical chronic inflammatory reaction and interfering the treatment success (16).

Root canal ramifications, such as lateral, secondary and accessory canals, can establish communication pathways between the main root canal and periodontal ligament, as well as the apical foramen (17,18). Several authors have reported that localized periodontal problems might arise due to the presence of necrotic and infected root canal ramifications, indicating the importance of the penetration of endodontic sealers into these irregularities (17,19). Although this physical property is very important, no study has evaluated the relationship between the sealer’s flow and its ability to penetrate into narrow root canal ramifications (17,20).

-Microleakage assessment methodologies

-Methodologies that use dyes

The methodology that uses tooth immersion in various types of dyes (5% eosin, 0.5-2% methylene blue, 0.5-1% black India ink, blue ink, black ink, drawing ink, Procion brilliant blue, 0.5% Rhodamine B, 0.5% fuchsin and others) is easy to perform (21-30). The teeth are sectioned longitudinally or transversely or cleared and the linear penetration of the dye is recorded (31-33). The disadvantages of longitudinal dentinal sectioning seem to be the random nature of selecting the cut axis and the very low odds of the sections being cut through the deepest dye penetration point, resulting in underestimation of leakage and achieving unreliable data (34). Depth of penetration can be assessed under a stereomicroscope at ×4 to ×30 magnifications with all the dyes (35,36). Confocal laser scanning microscope can be used for fluorescent dyes like Rhodamine B (22,37), photography and calculation using the ImageJ program (38).

Thermocycling or mechanical cycling have been used in some microleakage studies to better simulate clinical situations (39).

-Transverse sectioning to assess apical microleakage

The whole surface of each root was coated with two layers of nail varnish with different colors except for 3 mm from the root apex. The teeth were then immersed in a 50 wt% of silver nitrate (NO3Ag2) (40) solution for 1 hour in a dark environment, follo-wed by rinsing under distilled running water for 1 minute to eliminate the silver ions from the surface. Then, the teeth were immersed in a photo-developing solution and exposed to light for 12 hours. The teeth were then rinsed in distilled water and the roots were sectioned transversely at 1-mm intervals using a low-speed diamond disc. The sections resulted in three slices labeled A, B and C and considered first, second and third based on their distance from the apex. Each slice was divided into 4 equal parts and evaluated under a stereomicroscope at ×30. Dye penetration was scored as 0, 1, 2, 3 or 4 based on the amount of microleakage (1).

The clearing technique allows a 3D view of the internal anatomy of root canals with no loss of the tooth substance, facilitating visualization of the leakage area. It is a simple and fast technique, with no need for complex equipment (41). The technique facilitates observation of the lateral and accessory canals (31,42,43), clearly revealing the relationship between the sealing material and the apical foramen. The disadvantage is deficient demineralization (44), compromising the final transparency of the specimen. This technique is more accurate than the transverse sectioning for detecting apical leakage since it reveals leakage in fractions of millimeter, while transverse sectioning only determines the presence or absence of leakage (21).

-Dye selection

In relation to dyes, particle molecular size, pH and chemical reactivity affect the degree of penetration (32). A large number of studies have used methylene blue as a dye (28,34,45) because it is inexpensive, easy to handle, has a high degree of staining (46) and has a molecular weight lower than that of bacterial toxins (47). Some researchers have suggested that methylene blue exhibits leakage similar to butyric acid (48), a microbial metabolic product with greater penetration than Indian ink. This dye has some disadvantages such as dissolution during the demineralization and clearing processes; in addition, it is difficult to observe its maximum penetration point in some cases (49). A number of studies have suggested that Rhodamine B be used instead of methylene blue (50). One study reported that drawing ink exhibited less penetration into the root dentin compared to all the others (24). The particle size of this dye (0.1-2 µm) is comparable to the size range of a number of endodontic pathogens and appears to be advantageous in endodontic dye leakage studies (24). On the other hand, Barthel *et al.* (51) propounded that the particle size of the dye might not be an important factor in leakage studies.

India ink particles with diameters ≤3 μm are also widely used (36,49). It has been reported that the weight and size of India ink molecules are less than those of bacterial components isolated from the root canals. Therefore, it might not precisely simulate the molecules of fluids originating from periradicular tissues, yielding false-positive results during leakage studies (52).

An important consideration in relation to dye penetration studies is that air trapped in voids within the root canal obturation material might interfere with fluid movement. It has been recommended that dye penetration studies should be carried out under reduced pressure, erroneously referred to as vacuum (53). However, it is difficult to eliminate the trapped air by applying low pressure to small empty spaces, including those measuring 2 μm in diameter, which are permeable to bacteria (53). Kontakiotis *et al.* (45) investigated the influence of hydration on voids within the root canal obturation materials using a fluid transport model and dye penetration, in which air was applied to eliminate water from the voids in one group and showed that methylene blue penetrated more easily into dry gaps than into water-filled gaps. Methylene blue penetrated along air-filled gaps by capillary action, whereas it penetrated into water-filled gaps by diffusion (21). It has been reported that the vacuum method gave rise to significantly more dye penetration compared to fluid filtration and passive dye penetration (54).

-Fluid filtration or transportation method

In this method, the sealing ability is measured via air bubble movement within a capillary tube (21,55-58), consisting of an obturated canal with its coronal portion connected to a tube filled with water at atmospheric pressure, and its apex connected to a 20-μL glass capillary tube measuring 170 mm in length and with a uniform caliber filled with water. A pressure of 0.1 atm is applied through the coronal part, forcing the water through the empty spaces along the root canal (53). The results are reported in μL/min (59). The air bubble movements can be observed by computer-controlled diode laser beams rather than visually (21). Apical leakage can be measured with the use of a computerized fluid filtration meter consisting of a laser system (60).

-Dye extraction or dissolution method

In this method, the teeth are dissolved in acids, releasing all the dyes from the interfacial areas and a spectrophotometer determines the optical density (OD) of the solution (21).

-Methodology: Artificial saliva for 24 hours, placed in 2% methylene blue dye for 72 h. Then the specimens were washed with distilled water, the nail varnish removed, and then they were placed in 35% nitric acid for 72 hours. Standard solutions of 1%, 0.5%, 0.005%, 0.002%, 0.001%, and 0.0005% of methylene blue in 35% nitric acid were prepared and stored for 72 hours. At the end of this time period, the liquid was centrifuged for 1 minute (200 rpm) and the supernatant was subjected to spectrophotometric analysis at 670 nm to analyze the amount of leakage (61).

There was no correlation between dye penetration and the fluid filtration and dye extraction techniques which determine micro-leakage (34). The fluid filtration technique yielded results similar to those of dye extraction because both consider the porosity of the interface between the obturation material and the root (21). Both techniques rely on quantitative measurements of liquids passing through these interfaces (21). The dye extraction technique is superior over the fluid filtration method because the filtration values tend to diminish over time as water penetrates all the irregularities until a plateau is reached (21).

-Bacteria and toxin infiltration method

Use of bacterial organisms for the evaluation of leakage (mainly coronal) is believed to be more relevant clinically and biologically compared to the dye penetration method. A wide variety of bacterial strains have been used to assess marginal leakage, yielding contradictory results, because the methods depend on the type of bacterial strains used. In addition, if the sealer exhibits antimicrobial activity, it is not possible to employ the bacterial method (49,62). The systems generally consist of two chambers, making it possible to completely separate the apical and coronal ends of each specimen. The turbidity of the apical chamber broth is the first indication of bacterial contamination (63-65). If the pulp chamber becomes contaminated, it might be a reservoir for microorganisms and toxins, giving rise to a problem in either of the two ways. First, the apical seal might be affected, resulting in the failure of the root canal treatment. Second, penetration of microorganisms and toxins through the accessory canals on the pulp chamber floor might lead to periodontal furcation involvement (66). Bacterial studies are considered qualitative rather than quantitative (21). If only one bacterium penetrates into the obturated root canal, it might multiply in the enriched broth, causing turbidity (65,66). Different microorganisms have been tested to this end, including *Staphylococcus epidermidis* (51), *Enterococcus faecalis* (64,67-69), *P. mirabilis* (70), *Candida albicans* (71), *Streptococcus salivarius* (62), *S. epidermides* (70), *Candida albicans* (71), *S. mutans*, *S. mitis*, *Prevotella melaninogenica*, and *Lactobacillus acidophilus* (71), *Actinomyces odontotylicus*, *Lactobacillus acidophilus*, and *Pseudomonas fluorescens* (72), *anaerobic Streptococci* and *Fusobacterium nucleatum* (66) and the human whole saliva (63). *Enterococcus faecalis* is the most frequently used bacterial species. Penetration of bacteria or bacterial products might initiate or reactivate the inflammatory process (21). The molecular size of the test material must correspond to the bacteria and/or bacterial cell wall components and/or nutrient fluids. Leakage evaluation of root-end filling materials with the use of endotoxins is another method (73). It has been reported that endotoxins penetration preceded that of bacteria into the canal system (74).

-Glucose penetration model using fluid filtration 

A new method for the analysis of endodontic microleakage has been introduced that relies on the filtration rate of glucose along the root canal obturation material (75-77). In this technique, leakage is quantified with spectrophotometry (21). Another quantitative method is to measure the concentration of leaked glucose in the apical reservoir at different time intervals with the use of enzymatic glucose oxidase method (77).

Glucose was selected as a tracing agent due to its small molecular size (MW = 180 Da) and because it is a nutrient for bacteria (21). Therefore, if glucose penetrates into the root canal from the oral cavity, the bacteria surviving the root canal preparation and obturation procedures can multiply, causing periapical inflammation (21). Glucose leakage model (GLM) is considered a very sensitive and clinically relevant sealability test (77) compared to other leakage tests. However, its disadvantages include the long experimental period, the difficulty in maintaining a bacteria-free system to prevent consumption of glucose and the risk of water evaporation.

Pilot tests are necessary to assess glucose reactivity. According to Bernabé *et al.* (78), an approximate experimental time of 60 minutes and vacuum were selected in order to make sure that no glucose reaction would take place and also to facilitate the penetration of the marker. However, this glucose reactivity time can be affected by the type of the material tested and it is better to repeat it for each experiment.

-Methodology: A double-chamber should be assembled to measure glucose leakage. The maxillary segment of the syringe containing the glucose solution is connected to a pressure source to create a headspace pressure of 103 kPa per 60 minutes (76). Souza *et al.* (79) amplified the pressure in the maxillary chamber of the GLM system to accelerate glucose leakage from weeks to hours in order to decrease the risk of bacterial growth and long-term water evaporation, while maintaining the capacity to detect the leaking samples.

Michailesco *et al.* (72) used latex microspheres and showed that they can be used to simulate bacterial leakage and that smaller particles penetrate deeper than bacteria and vice versa.

-Protein microleakage test

Protein microleakage test is carried out using bovine serum albumin in a dual-chamber apparatus and calculated using a spectrophotometer (80-82).

Methodology: In order to evaluate leakage, bovine serum albumin marker is traced in a dual-chamber apparatus with Bradford indicator. Bradford indicator is used to determine the concentration of albumin leaking from the upper chamber into the lower chamber. The procedure relies on the formation of a complex between the dye, Brilliant Blue G, and proteins in solution. The subsequent formation of the protein-dye complex shifts the wavelength of maximum absorption of Coomassie Brilliant Blue G from 465 to 596 nm. The absorption rate is proportional to the protein present (82).

Caffeine microleakage is another method with the use of a modified dual-chamber model; epoxy resin is used for sealing of the margins instead of cyanoacrylate glue and leaked caffeine content can be detected by high-performance liquid chromatograp-hy-tandem mass spectrometry (HPLC-MS/MS) (83) or a visible ultraviolet spectrophotometer (UV-VIS detectors) (77,84). The caffeine detection limit was 2000 ng mL-1, using UV-VIS, and 10 ng mL-1, using HPLC-MS/MS. HPLC-MS/MS can detect concentrations at least 1000 times lower (83).

-Electrochemical microleakage test

The electrochemical method relies on the diffusion of ions through very narrow spaces, with the results possibly depending on electrical laws (85). It is believed that the magnitude of the electrical current produced by ions diffusion, between two electrodes, is directly proportional to the amount of leakage. Changes in ion concentration can affect the results. Another parameter that can be measured in the electrochemical test is the electric resistance. Resistance and leakage are inversely related to each other. As the leakage increases, the electric resistance decreases.

-Methodology: The external surfaces of the teeth were completely covered with nail varnish, sparing the access opening and the apical foramen. The roots were mounted in silicon through the bottom of plastic cylinders, leaving the access open within the cylinder. The cylinders were filled with saline solution to serve as an electrolyte. The cylinders in association with the teeth were mounted in Petri dishes filled with the saline solution as an electrolyte. Only 2 mm of the root ends were immersed in the solution. To carry out measurements, a #70 K-file was placed in each upper chamber and a stainless steel wire was inserted into the Petri dish. The electrode in each upper chamber was separately connected to the electrode in the lower chamber through an electric circuit with an 8-V direct current power supply. The electrical conductivity in this circuit was measured in μA for each root.

Some researchers (86) applied two different types of metal as electrodes (stainless steel and copper). This procedure may lead to an electrical potential that creates between two electrodes and effects on our measurements. The results of electrochemical microleakage tests were very divergent. This may be partly attributed to differences in the composition of the electrolyte, electrode type and distance between the two electrodes, electrode thickness and electrical conductivity of the ionic solution.

-The radioisotope penetration method 

The radioisotope penetration method is a qualitative method (87). In the analysis of results; its two-dimensional autoradiograph image is not representative of the three-dimensional image of micro-leakage. An isotope, such as Ca, has an affinity to tooth structure or restorative materials leading to increased measurement errors. Moreover, isotopes can pass through tooth structure or restorative material flaws as a result of their tiny size; resulting in misinterpretation of leakage (88).

Copper ion has been introduced as a new leakage tracer. Copper sulfate diffusion method was used since copper ions are not naturally detected in tooth structures by conventional methods (87). CuSO4 solution was injected into the coronal segment of the specimens with a fine needle. After two days, the copper sulfate concentration in the solution was measured by an atomic absorption spectrometer (87).

-Animal experiments

Animal studies on dogs undergoing endodontic treatment with a minimum 8 months of follow-up is another technique, with ra-diographs being taken every 2 months and histologic examinations after completion of the follow-up period (89). More extensive and long-term studies are necessary to establish a better and more efficient system for microleakage studies.

-Three-dimensional method

CBCT (90) and SEM evaluation (91) can be used for evaluation of maximum and minimum amounts of voids and dentin diffusion of sealer in obturation materials and can be representative of leakage potential of endodontic materials.

Bond strength, push-out bond strength and bond durability (92) can reveal leakage potential of endodontic materials.

## Conclusions

The complexity of the subject resulted in a reduced number of articles on “obturation and microleakage”. However, it is important to obtain a tight seal in obturated root canals and it is necessary to conduct clinical or laboratory studies to assess the sealability of endodontic materials. The combination of all innovative technologies has allowed the clinicians to render the best care available and achieve the most predictable outcomes possible.

Therefore, the clinicians should adopt these technologies and use them in the most predictable and efficient way possible. Thus, prevention of microleakage paves the way for more predictable and successful endodontic outcomes.

All the methods mentioned above are useful if the study is performed strictly with a large sample size and proper control groups. However, further studies should be performed on the clinical relevance of microleakage tests and their reliability and correlation between different leakage assessment methodologies.
